# Novel PSMA targeting alpha-emitting radioligand [^211^At]PSAt-3-Ga inhibits tumor growth and increases survival in a preclinical model of human xenograft prostate cancer

**DOI:** 10.1186/s13550-026-01378-z

**Published:** 2026-01-27

**Authors:** Lars Hvass, Marius Müller, Vladimir Shalgunov, Anne S. Clausen, Christian B. M. Poulie, Emma Aneheim, Holger J. Jensen, Matthias M. Herth, Andreas Kjaer

**Affiliations:** 1 Cluster for Molecular Imaging, Department of Biomedical Sciences, University of Copenhagen, Blegdamsvej 3, Copenhagen N, 2200 Denmark; 2 Department of Clinical Physiology, Nuclear Medicine & PET, Rigshospitalet, Blegdamsvej 9, Copenhagen, 2100 Denmark; 3 Department of Drug Design and Pharmacology, Faculty of Health and Medical Sciences, University of Copenhagen, Universitetsparken 2, Copenhagen, 2100 Denmark; 4 Department of Radiation Physics, Institute of Clinical Sciences, Sahlgrenska Academy, University of Gothenburg, Gothenburg, Sweden

**Keywords:** Prostate cancer, Targeted radioligand therapy, Astatine-211, PSMA, Alpha therapy

## Introduction

Prostate cancer is one of the most common cancers in men with more than one million new cases diagnosed annually [1, 2]. Prostate cancer diagnosed at a localized stage of disease may be treated by several approaches including surgery and external beam radiation therapy, or surveilled if not requiring immediate treatment [3]. Nonetheless, many patients progress to metastatic disease, at which point androgen deprivation therapy (ADT) in combination with chemotherapy is employed [4, 5]. Despite initial disease control, selection pressure for castration resistant cancer cells often lead to castration resistant prostate cancer (CRPC) and progression [6]. Metastatic CRPC (mCRPC) remains challenging to treat and has a poor prognosis with a median survival of 2–3 years [7]. Despite continued progress, better treatment options for metastasized disease are warranted to improve patient outcome.

For this, targeted radioligand therapy (RLT) is gaining prominence in the treatment of mCRPC. As such, the beta-emitter [^177^Lu]Lu-PSMA-617 gained FDA approval in 2022 based on convincing therapeutic efficacy in patients with mCRPC [8]. This treatment is administered after verifying PSMA expression in malignant tumors by PET imaging and thus deliver localized beta radiation therapy at site of disease. Furthermore, the treatment has shown clinical benefit in taxane-naïve patients [9]. Recently, analogous approaches using alpha emitters have been suggested as a means to improve efficacy, and actinium-225 (^225^Ac) has shown encouraging effect in patients progressing on [^177^Lu]Lu-PSMA-617 [10, 11]. Alpha particles boast many fold higher linear energy transfer relative to beta particles, as well as a markedly shorter maximum range in tissue [12]. As such, these characteristics are considered favorable for targeting disseminated disease with limited off-target toxicity and high dose deposition in targeted cells [13, 14]. Of these, astatine-211 (^211^At) is deemed especially suitable due to a decay chain containing only one alpha emission (^225^Ac has four) omitting recoiling daughters [15] as well as a short half-life (7.2 h) suitable for peptide labelling while reducing waste management concerns [15].

**Fig. 1:**
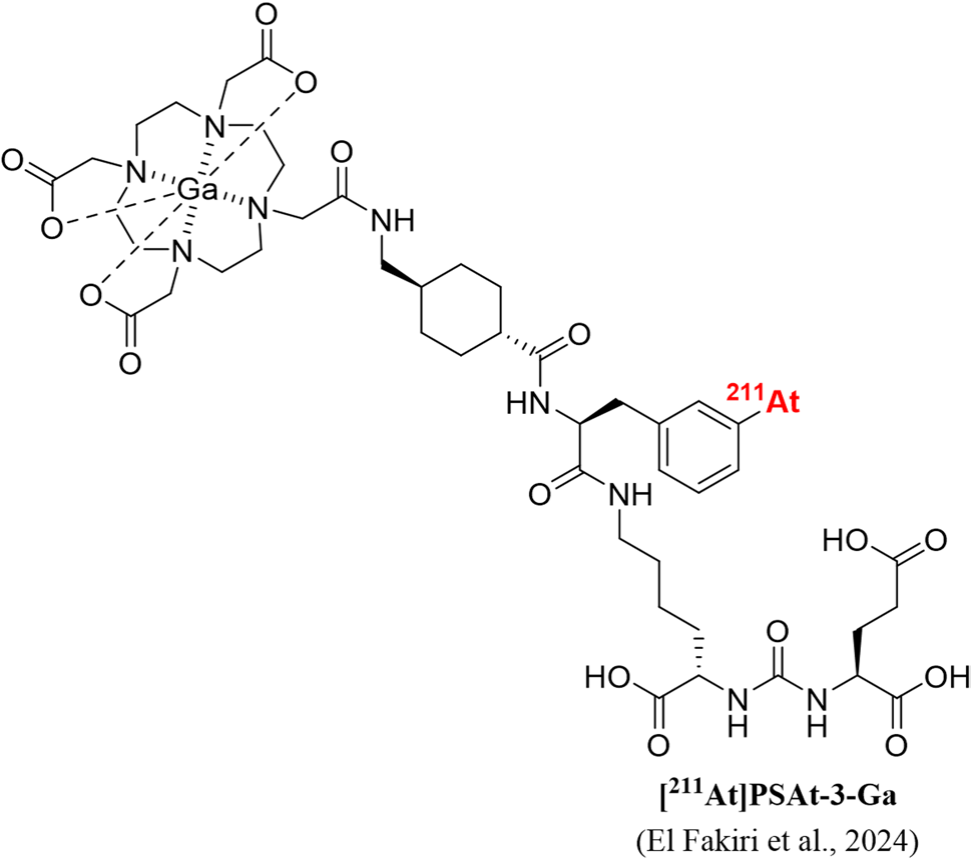
Structural depiction of PSAt-3-Ga

We have previously published encouraging biodistribution data of a novel radioligand for prostate cancer treatment, [^211^At]PSAt3-Ga (Fig. 1), a structural derivative of PSMA-617, labelled with the alpha emitter ^211^At [16]. We demonstrated high tumor retention, acceptable kidney retention, and a low degree of deastatination was evident. Other studies investigating novel [^211^At]-labeled compounds have evaluated doses ranging from 0.32 to 3.7 MBq [17, 18]. In this brief communication, we hypothesize that a moderate dose of 0.5 MBq [^211^At]PSAt3-Ga is sufficient to inhibit tumor growth and increase survival in a xenografted mouse model of human prostate cancer with low systemic toxicity.

## Method

### Radiochemistry

^211^At was produced by ^209^Bi(alpha,2n)^211^At reaction on a scanditronix MC32 cyclotron at Copenhagen University Hospital and isolated by dry distillation (Atley C100, Atley solutions), after which it was dissolved in chloroform. [^211^At]PSAt-3-Ga was then produced as previously described with a radiochemical conversion of approx. 30% [16] (SI Figs. 1, 2 and 3). [^68^Ga]Ga-PSMA-617 was produced as described in supplementary information (SI).

### In vivo efficacy

Inbred male BALB/cAnN-*Foxn1*^*nu/nu*^/Rj mice were acquired from Janvier (France) and housed at University of Copenhagen in autoclaved and individually ventilated cages in a 12:12 h light/dark cycle with ad libitum access to food and water. After a one week acclimatization period, subcutaneous xenografts were established by inoculation with 3–4 million LNCaP cells (CRL-1740; ATCC) in a 1:1 mixture of phosphate-buffered saline and Corning Matrigel Matrix (USA). Cell culture was maintained according to ATCC recommendations, with the addition of 1% penicillin-streptomycin to avoid bacterial infection. Cells were authenticated by short-tandem repeat sequencing (ATCC). Approximately three weeks after inoculation, mice were randomized into two groups (treatment *n* = 7, and control *n* = 11) based on tumor size (129 ± 47 and 128 ± 52 mm^3^, respectively. One group was intravenously injected in a tail vein with 0.47+−0.03 MBq [^211^At]PSAt3-Ga (*n* = 7), and a control group with saline (*n* = 11). Animals were monitored daily for signs of discomfort, and tumor size monitored three times weekly by caliper measurement. Endpoints were tumor size = 10% bodyweight or 2 cm^3^. Humane endpoints were 20% bodyweight reduction or signs of discomfort. To assess hematological toxicity, 25 uL blood samples were collected from vena saphena of representative animals (*n* = 4 and 8 for treated and control, respectively) in EDTA and blood composition analyzed using an Element HT5 hematology analyzer (Heska, USA).

### PET/CT imaging

Upon tumor growth resumption in treated animals, 5.6 ± 1.2 MBq [^68^Ga]Ga-PSMA-617 was injected intravenously in a subset of treated (*n* = 4) and control (*n* = 5) animals and PET/CT scanned using a Siemens Inveon PET/CT scanner (USA) one hour after injection. This was to infer whether PSMA-dependent radioligand accumulation was suppressed after radioligand therapy. See SI for scan parameters.

### Immunohistochemistry

Reaching endpoint, all mice were euthanized and tumor, salivary glands, and kidneys fixated in 4% PFAwere embedded in paraffin for all except one control animal. Slices of salivary glands and kidneys were H&E stained and examined for aberrations. Tumor sections were stained for PSMA (Abcam, AB133579). Stains were then digitalized (Zeiss Axioscan 7 slide scanner) at 10x magnification. Subsequently, %PSMA-positive tumor area was determined by training and using QuPath pixel qualifier [19].

### Statistics

Survival probability was compared using Mantel-Cox log-rank test. All other comparisons were carried out using Welch’s t-test. All analyses were conducted in R version 4.3 and graphs produced in Graphpad Prism or R using the Survminer package [20].

## Results

### In vivo efficacy

**Fig. 2:**
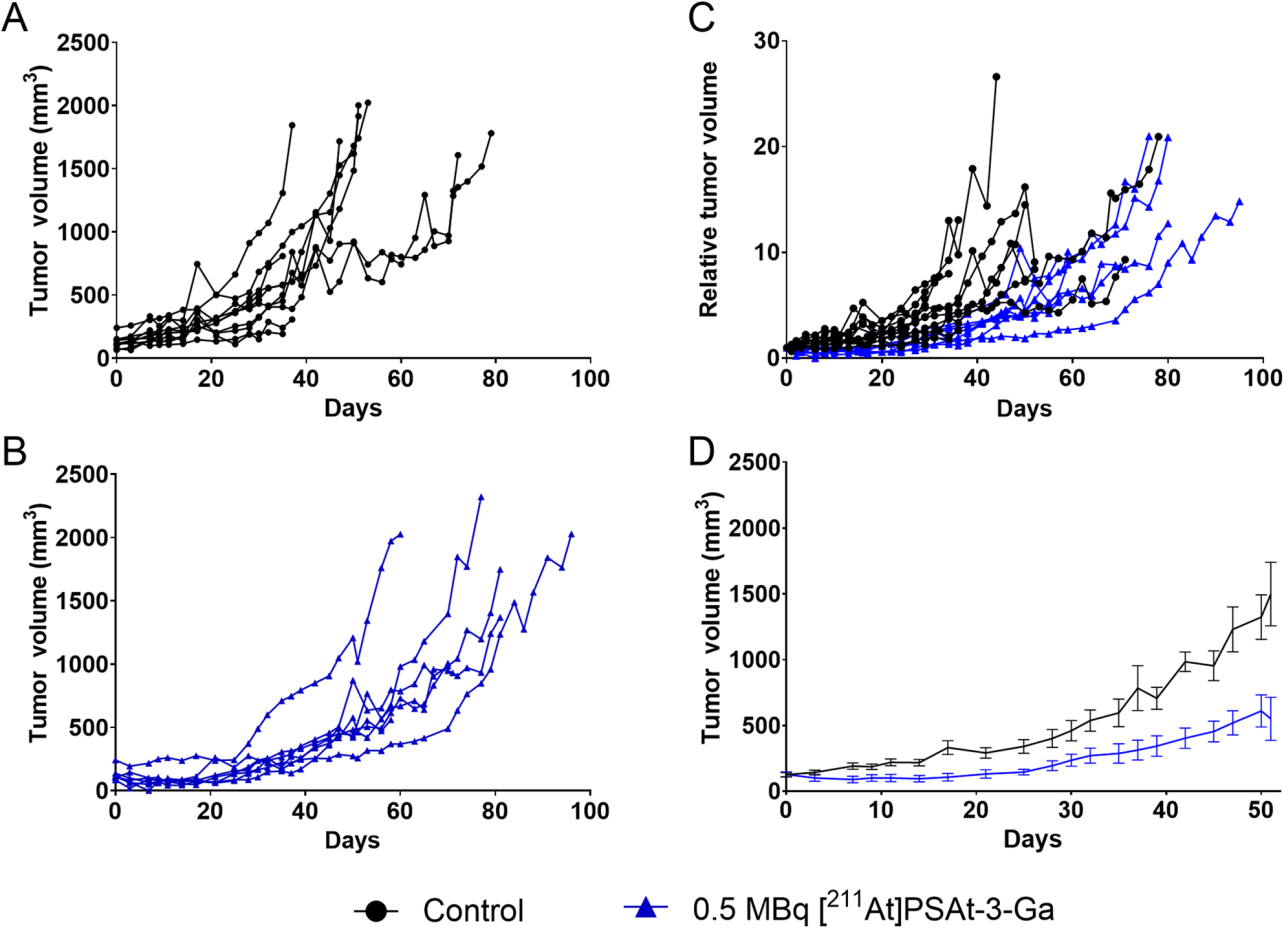
Tumor growth curves. (**A**) Tumor growth of saline treated control mice (*n* = 11). (**B**) Tumor growth in mice treated with 0.5 MBq [211At]PSAt3-Ga (*n* = 7). (**C**) Tumor growth normalized to initial tumor volume for both groups. (**D**) Average tumor growth in both groups until 50 days after treatment. Blue indicates treated, and black control animals

**Fig. 3:**
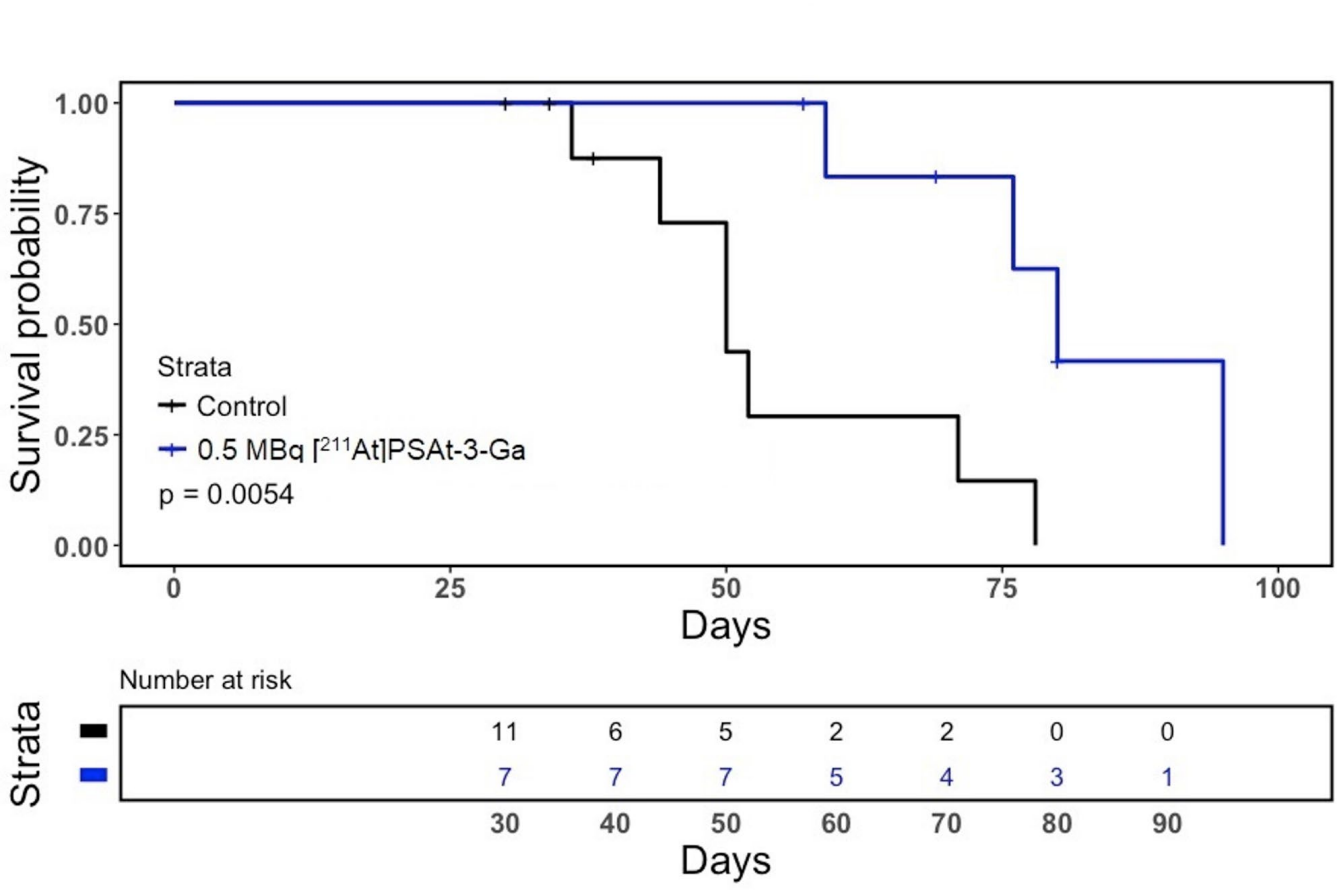
Survival Top) Kaplan-Meier survival curve depicting control (black) and treated (blue) groups survival probability vs. time (*P* =.0054). Bottom) Number at risk for the two strata

Intravenous treatment with one dose 0.5 MBq [^211^At]PSAt3-Ga reduced tumor size and delayed tumor growth in LNCaP xenografted mice (Fig. 2). Median time to endpoint (survival [humane endpoints]) increased from 50 to 80 days with treatment (*p* =.0054), demonstrating that 0.5 MBq [^211^At]PSAt3-Ga was sufficient to increase survival (Fig. 3).

### PSMA expression and toxicology – potential for retreatment

**Fig. 4:**
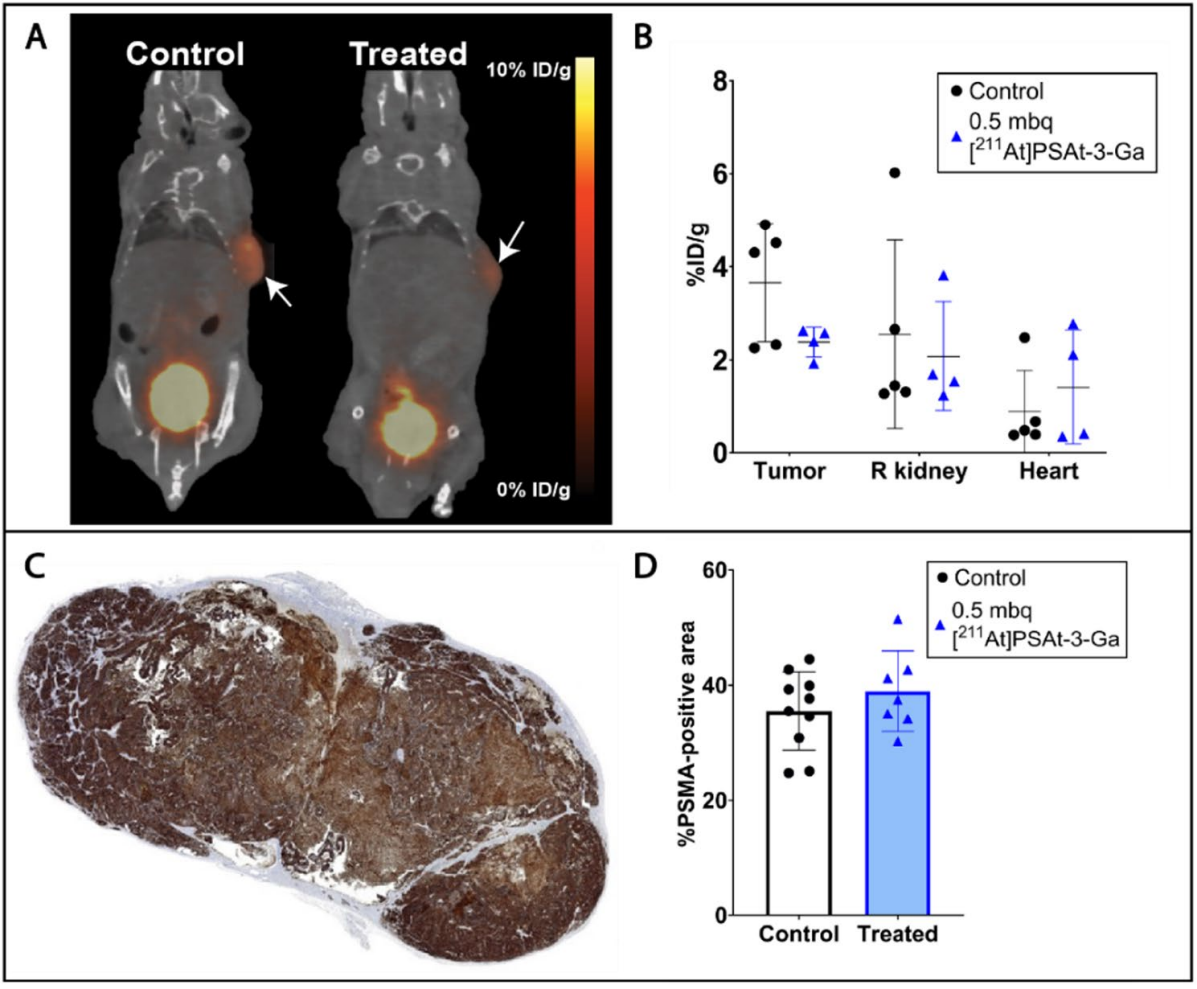
PSMA expression. (**A**) [^68^Ga]Ga-PSMA-617 PET/CT of a control (left) and a 0.5 MBq [^211^At]PSAt3-Ga treated (right) mouse after tumor growth resumption in treated mice. Arrows indicate tumors. (**B**) Quantification of PSMA PET scans in tumor, kidney, and heart in a subset of control (*n* = 5) and treated (*n* = 4) mice. (**C**) Section of tumor tissue stained immunohistochemically for PSMA. (**D**) %PSMA-positive tumor area in tumors from control (black circles, *n* = 10)) and treated (blue triangles, *n* = 7) mice euthanized at endpoint

Upon tumor growth resumption, PET imaging revealed a tendency toward reduced albeit non-significant (*p* =.0867) tumor accumulation of [^68^Ga]Ga-PSMA-617, while kidney and heart, used as a blood surrogate, concentration remained unaltered (Fig. 4 and SI Fig. 4). Contrarily, PSMA % positive area determined by immunohistochemistry at endpoint was unaltered in resected tumors of treated mice (*p* =.3274) (Fig. 4). Together, this indicates continued PSMA expression after treatment, but the level of expression may be lowered, as evident by lower quantitative PET uptake.

**Fig. 5:**
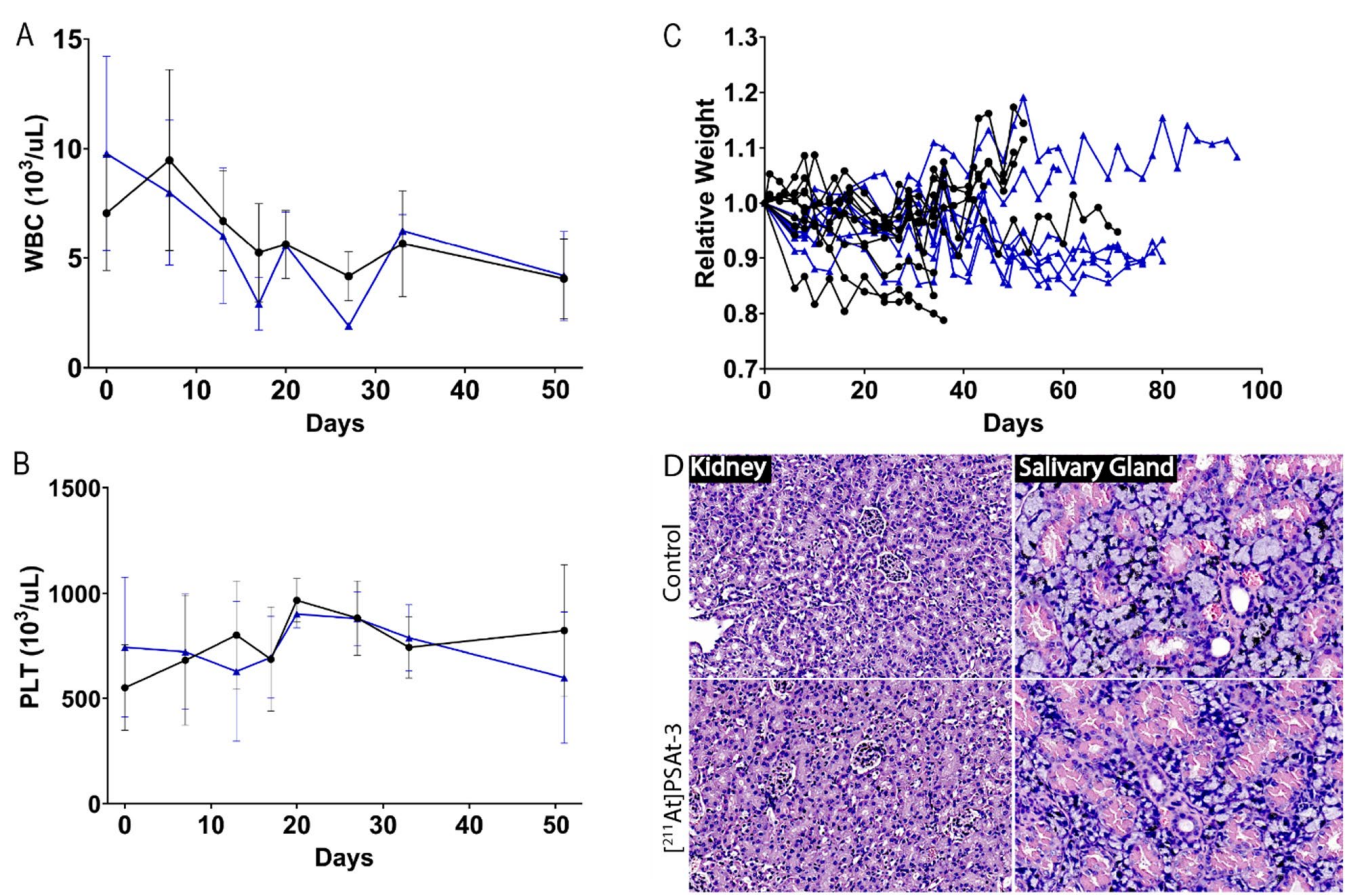
Toxicology. (**A**) White blood cells (WBC) and (**B**) Blood platelets (PLT) for a subset of *0.5 MBq [*^*211*^*At]PSAt3-Ga* treated (*n* = 4) and control (*n* = 8) mice during the first 50 days after treatment. (**C**) Relative weight of all animals from randomization to endpoint. (**D**) Representative H&E stains of cortical area of kidney and salivary gland. Blue indicates treated, and black control animals

To assess toxicology, a histopathological examination of kidney and salivary gland was conducted after mice had reached endpoint criteria. This analysis did not reveal any aberrations suspected from alpha irradiation such as dilated proximal tubules, inflammation, or necrotic areas (Fig. 5). Hematological assessment revealed a decline of white blood cells (WBC) over the course of therapy which was equally apparent in control animals. However, no animals were observed to have leukopenia. Treatment had no apparent effect on blood platelets (PLT) (Fig. 5). Both parameters have previously been associated with transient reductions during alpha radiation therapy by ^225^Ac [11]. For all hematologic parameters, please see SI Fig. 5.

## Discussion

The treatment effect of [^211^At]PSAt-3-Ga on tumor growth and survival time presented here corroborate conclusions from other studies targeting PSMA with similar urea-based binding motifs for delivery of ^211^At radiation to prostate cancer cells [17, 18, 21]. Of note, Watabe and colleagues report shorter general survival times in treated and untreated animals, despite using the same xenograft model [21]. This discrepancy may be partly due to the combination higher inoculation load and the use of NOD/SCID mice, which show a higher rate of tumorigenesis and growth rate of slow-growing tumors [22]. Direct comparison of efficacy with [^211^At]−3-Lu and [^211^At]At-NpG-PSMA [17, 18] is difficult as a different model and a cell line engineered to vastly overexpress PSMA was employed in evaluation of these compounds.

A similar dose to kidney can be inferred from renal accumulation of [^211^At]PSMA5 based on biodistribution [21]. In this study, Watabe et al. found indications of regenerated tubules in the kidney 3 and 6 weeks after administration of 0.4 MBq [^211^At]PSMA5 in NOD/SCID mice. Absence of renal damage may be attributable to a faster observed renal washout of our compound, [^211^At]PSAt3-Ga, compared to [^211^At]PSMA5, as well as lower radiation tolerance of NOD/SCID mice relative to the BALB/c strain used in this study [23].

Maximum tolerable dose was not reached in this study, which in combination with continued PSMA ligand accumulation and PSMA expression indicates the potential for a repeated dosing regimen.

## Conclusion

A single dose of 0.5 MBq [^211^At]PSAt-3-Ga effectively inhibited tumor growth and increased median survival in a xenografted model of human prostate cancer without any toxicity. Pending further experiments with repetitive treatment, mimicking the clinical use case, we suggest [^211^At]PSAt-3-Ga as a potential candidate for targeted alpha-therapy of metastatic prostate cancer in patients.

## Supplementary Information

Supplementary Material 1

## Data Availability

data used in this study is available from the corresponding author on request.

## Abbreviations

ADT: Androgen Deprivation Therapy
CRPC: Castration Resistant Prostate Cancer
mCRPC: Metastatic Castration Resistant Prostate Cancer
RLT: Radioligand Therapy
PSMA: Prostate-Specific Membrane Antigen
SI: Supplementary Information
H&E: Hematoxylin and Eosin (staining method)
PET/CT: Positron Emission Tomography / Computed Tomography
WBC: White Blood Cells
PLT: Platelets
[^177^Lu]: Lutetium-177
[^68^Ga]: Gallium-68
[^211^At]: Astatine-211
[^225^Ac]: Actinium-225
