# Supplementary material for: Novel PSMA targeting alpha-emitting radioligand [211At]PSAt-3-Ga inhibits tumor growth and increases survival in a preclinical model of human xenograft prostate cancer
Source: EJNMMI Res. 2026 Jan 27;16:34. doi: 10.1186/s13550-026-01378-z (PMC12917014; doi:10.1186/s13550-026-01378-z)
Supplement: Supplementary file 1 — Supplementary Material 1 [file 13550_2026_1378_MOESM1_ESM.docx]

## Novel PSMA targeting alpha-emitting radioligand [^211^At]PSAt-3-Ga inhibits tumor growth and increases survival in a preclinical model of human xenograft prostate cancer

## Supporting Information

Lars Hvass^a,b^*, Marius Müller^,c^*, Vladimir Shalgunov^c^, Anne S. Clausen^a,b^, Christian B.M. Poulie^c^, Emma Aneheim^d^, Holger J. Jensen^b^, Matthias M. Herth^b,c#^, Andreas Kjaer^a,b#^

^a^Cluster for Molecular Imaging, Department of Biomedical Sciences, University of Copenhagen, Blegdamsvej 3, 2200 Copenhagen N, Denmark;  ^b^Department of Clinical Physiology, Nuclear Medicine & PET, Rigshospitalet, Blegdamsvej 9, 2100 Copenhagen, Denmark; ^c^Department of Drug Design and Pharmacology, Faculty of Health and Medical Sciences, University of Copenhagen, Universitetsparken 2, 2100 Copenhagen, Denmark; and ^d^Department of Radiation Physics, Institute of Clinical Sciences, Sahlgrenska Academy, University of Gothenburg, Gothenburg, Sweden.

*For correspondence or reprints, contact Prof. Andreas Kjær (*[*Akjaer@sund.ku.dk*](mailto:Akjaer@sund.ku.dk)*), Blegdamsvej 3, 2200 CPH N*

*First author: Lars Hvass, PhD. Student.* [*Lars.Hvass@sund.ku.dk*](mailto:Lars.Hvass@sund.ku.dk)*, +4520673082, Blegdamsvej 3, 2200 CPH N,*

**Contributed equally*

*First authors: Lars Hvass, PhD. Student.* [*Lars.hvass@sund.ku.dk*](mailto:Lars.hvass@sund.ku.dk)*, +4520673082, Blegdamsvej 3b, 2200 CPH N, and Marius Müller, PhD,* [*marius.muller@sund.ku.dk*](mailto:marius.muller@sund.ku.dk)*, +4555208960, Universitetsparken 2, 2100 CPH Ø.*

**[^68^Ga]Ga-PSMA-617 production**

[^68^Ga]Ga-PSMA-617 was obtained by mixing [^68^Ga]GaCl_3_ (265 – 275 MBq) in 0.1 M HCl and a 1 M ammonium acetate solution in a ratio of 1:5 (final pH = 4.6). After the addition of 5 nmol of PSMA-617 (2.6 µL of a 2 mg/mL stock solution in DMSO), the reaction was left at 95 Degrees Celsius over 5 min. The reaction was allowed to cool to room temperature for 2 min and the radiochemical conversion (RCC, 52.7 ± 56.1 %) was determined by reverse-phase HPLC. Subsequently, purification was carried out via solid phase extraction employing a OASIS HLB 60 mg cartridge, which was preconditioned with EtOH (10 mL), H_2_O (10 mL) and air (5 mL). For that, the reaction mixture was passed through the HLB cartridge, which was subsequently washed with H_2_O (7 mL). After the elution of [^68^Ga]Ga-PSMA-617 from the HLB cartridge with EtOH (1 mL), the eluted fraction was concentrated under continuous stream of nitrogen at 60 Degrees Celsius over 10 – 15 min. After the EtOH had been completely removed, [^68^Ga]Ga-PSMA-617 was redissolved in phosphate buffered saline (pH 7.4). The radiochemical purity of [^68^Ga]Ga-PSMA-617 was determined to be > 90 % by reverse-phase HPLC. Molar activities were calculated to be 28.3 ± 29.2 MBq/nmol.

**PET/CT imaging**

A representative subset of mice in the treatment and control groups were injected intravenously with 5.6±1.2 MBq [^68^Ga]Ga-PSMA-617 in 100 uL PBS and PET/CT scanned (Inveon, Siemens Medical Solutions, USA) one hour after injection. During image acquisition mice were anesthetized by breating 30% oxygen enriched air containing 3.5% isoflourane. CT parameters: 360 projections, tube voltage of 65 kV with exposure time of 440 ms. Reconstructions were carried out with 2x downsampling. PET images were acquired using a 3.438 ns timing window and an energy window of 350-650 kev. Up to four mice were scanned simultaneously and body temperature kept constant by an in-house bed system modified from Greenwood.(*1*) Sinograms were then reconstructed using an OSEM3D/SP-MAP algorithm with scatter and attenuation correction in Inveon acquisition workplace (Siemens Medical Solutions, USA). On reconstructed scans, regions of interest were manually drawn on tumors and activity concentration expressed as Percent Injected Dose per gram (%ID/g).

**Immunohistochemistry:**

Resected tumors were fixated in buffered 4% paraformaldehyde (pH 7.2) followed by tissue preparation and embedding in paraffin. Tumors were cut in sections of 4 µM and dewaxed through xylene and ethanol to tap water. For antigen retrieval, the sections were heat treated for 15 min in 10 mmol citrate buffer (pH 6). This was followed by a blocking step with Peroxidase-Blocking Solution (Agilent, S2023) and pre-incubation in 2 % BSA for 10 min. Sections were incubated with PSMA antibody (Abcam, AB133579) in a 1:200 dilution in 2% BSA for 24 hours at 4°C. For visualization, the sections were incubated with Envision+ system Anti-Rabbit (Agilent, K4003) for 45 min followed by incubation with DAB+ system (Agilent, K3468) for 10 min. Nuclear counterstaining was performed with hematoxylin.

Kidneys were fixated in buffered 4% paraformaldehyde (pH 7.2) followed by tissue preparation and embedding in paraffin. The organs were cut in sections of 4 µM and dewaxed through xylene and ethanol to tap water. Consecutive sections of all samples were stained with hematoxylin and eosin.

**HPLC analysis of [^211^At]PSAt-3-Ga**

**
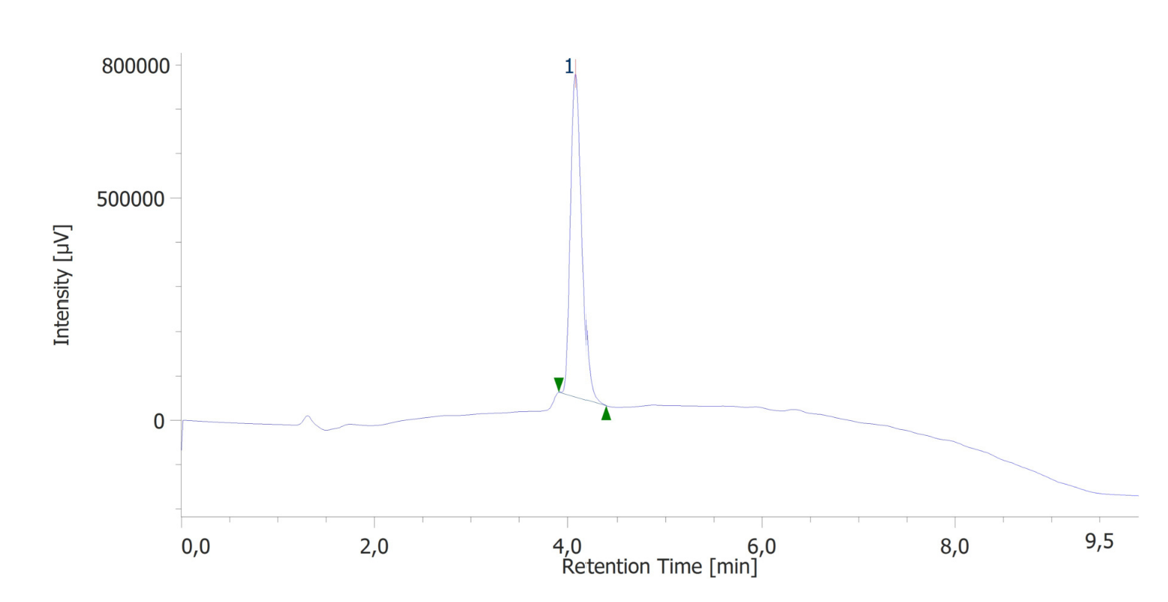
**

Supplementary figure 1: UV-HPLC chromatogram of the reference (Rt = 4.083 min) of [^211^At]PSAt-3-Ga.


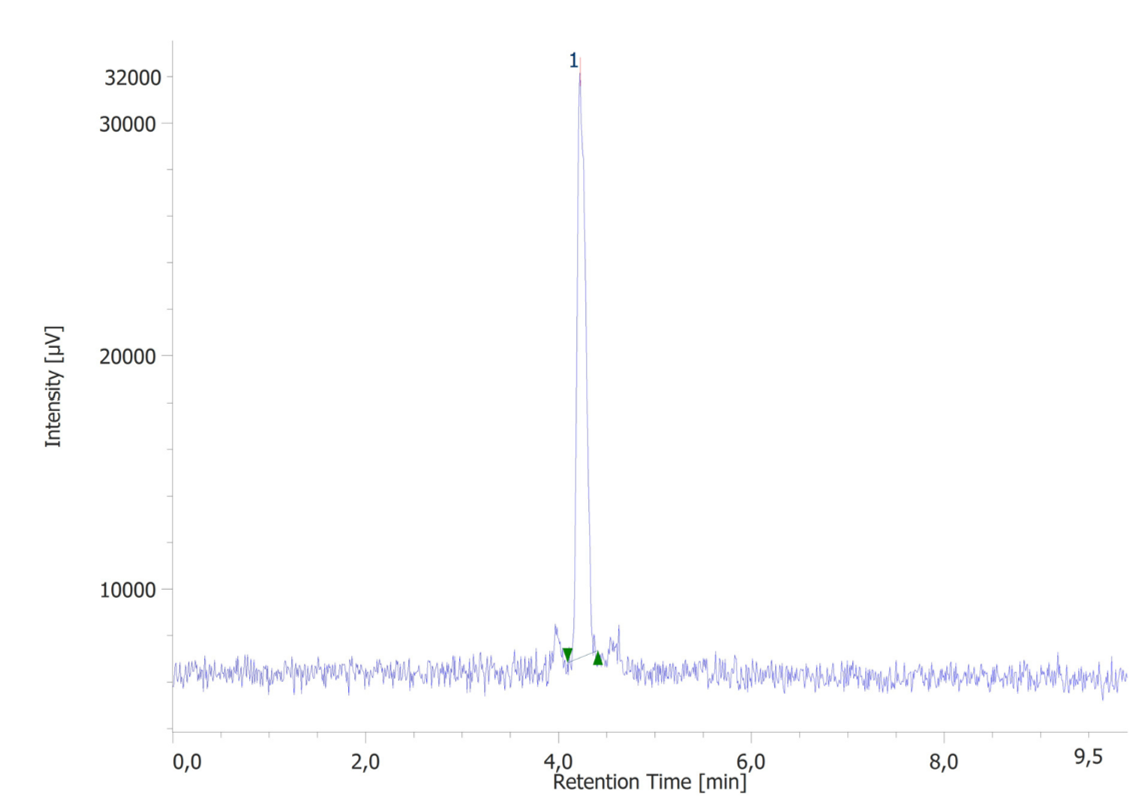


Supplementary figure 2: Radio-HPLC chromatogram of purified and radiolabeled [^211^At]PSAt-3-Ga (Rt = 4.225 min).


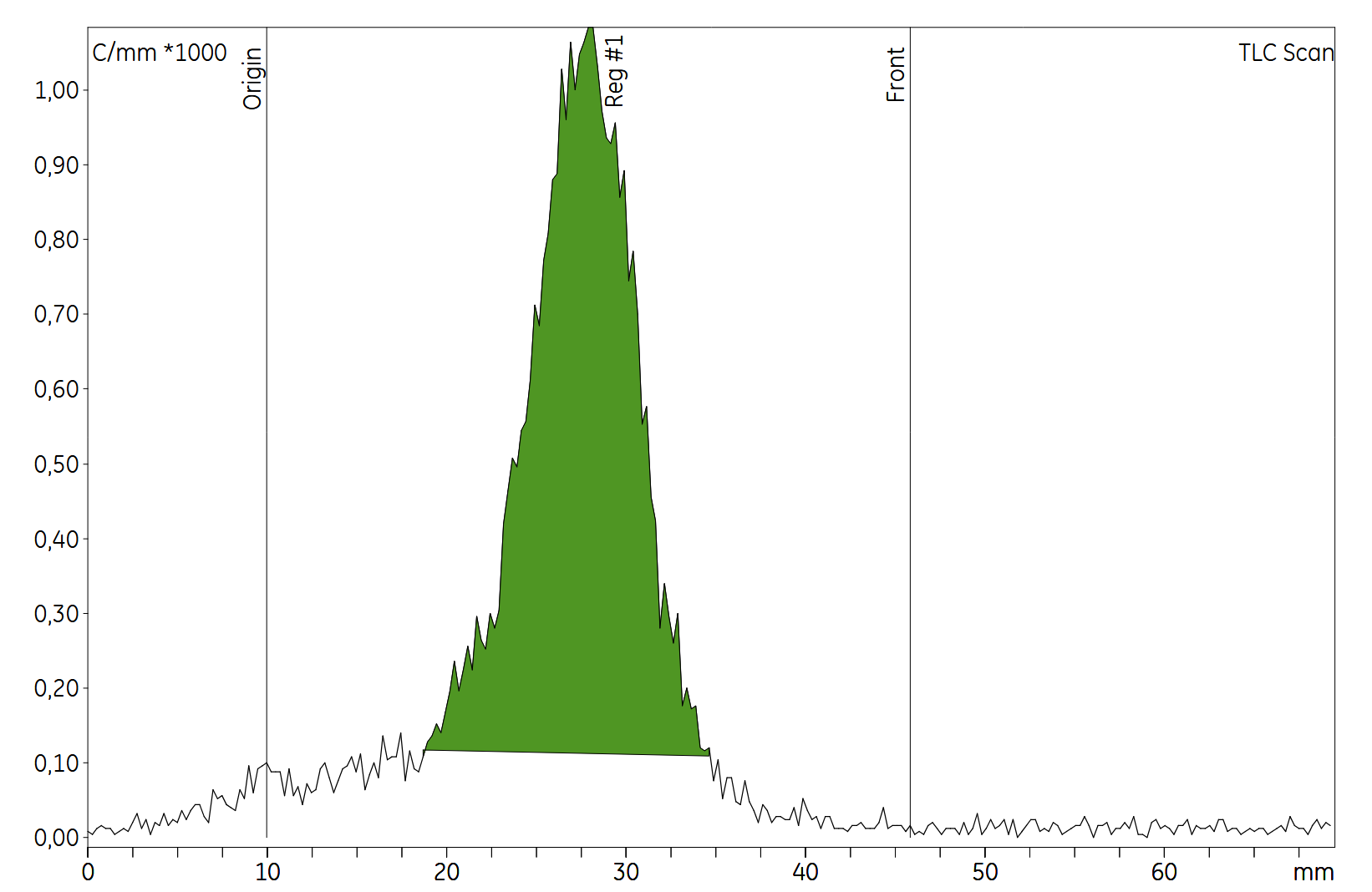


*Supplementary figure 3:* Radio-TLC of purified and formulated [^211^At]PSAt-3-Ga.

**Supplementary in vivo data**

**
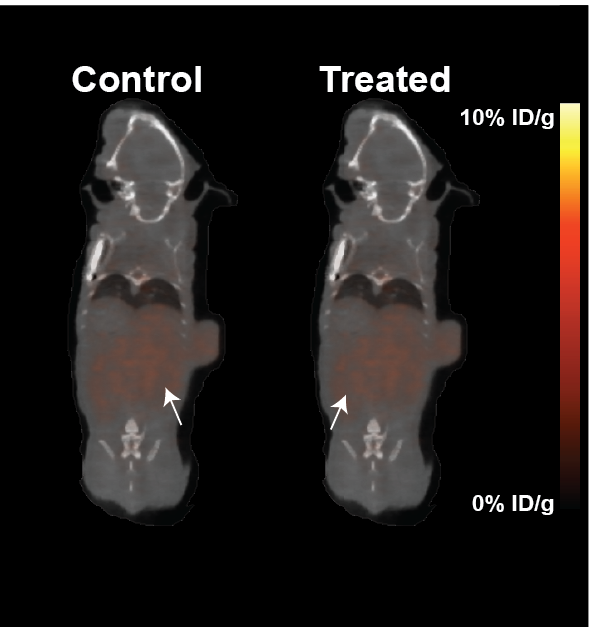
**

**Supplementary figure 4:** PET/CT scan one hour after injection with [^68^Ga]Ga-PSMA-617 of a representative control (left) and treated (right) mouse after tumor growth resumption in treated mice showing kidney accumulation. Arrows indicate kidney tissue.

**Hematology**

**
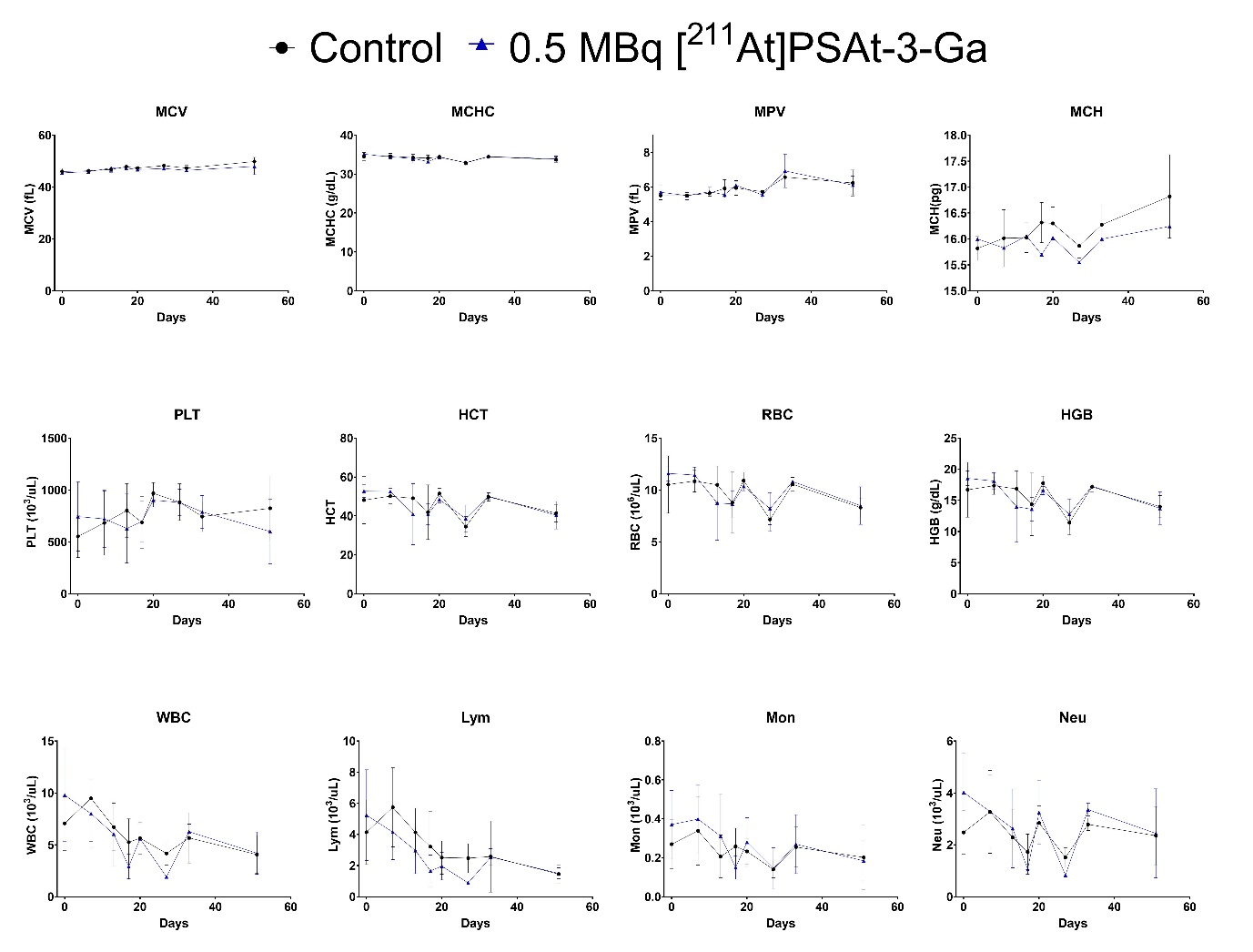
**

***Supplementary figure 5.*** *Hematological parameters. MCV (Mean Corpuscular Volume), MCHC (Mean Corpuscular Hemoglobin concentration), MPV (Mean Platelet Volume), MCH (Mean Corpuscular Hemoglobin), PLT (Platelets), HCT (Hematocrit), RBC (Red Blood Cells), HGB (Hemoglobin), WBC (White Blood Cells), Lym (Lymphocytes), Mon (Monocytes), Neu (Neutrophils)*

**1.** Greenwood HE, Nyitrai Z, Mocsai G, Hobor S, Witney TH. High-Throughput PET/CT Imaging Using a Multiple-Mouse Imaging System. *Journal of Nuclear Medicine.* 2020;61:292-297.
